# Assessment and management of allergic rhinitis: A review and evidence-informed approach for family medicine

**DOI:** 10.1002/jgf2.720

**Published:** 2024-07-14

**Authors:** Akash Jangan, Zahir Mughal, Ahmar Ahmad, Mark Simmons, Aziz Sheikh, Faraz Mughal

**Affiliations:** 1 Walsall Manor Hospital, Walsall, UK; 2 Usher Institute, The University of Edinburgh, Edinburgh, UK; 3 School of Medicine, Keele University, Keele, UK

**Keywords:** allergy, allergic rhinitis, nasal congestion, rhinorrhea

## Abstract

Allergic rhinitis is an inflammatory disorder affecting nasal mucosa in response to al-lergen exposure and is commonly assessed and managed in family medicine. In this article, we review new international guidelines on the diagnosis and management of allergic rhinitis and generate evidence-informed recommendations for family medicine doctors.

## 1 Introduction

Allergic rhinitis (AR) is a common condition encountered in family medicine, affecting up to 15% of the pediatric population and approximately 26% of adults in the United Kingdom.^1^ Although often mild, its impact can be far-reaching, affecting school and work performance.^1^ AR is characterized by an immunoglobulin E (IgE)-mediated type 1 hypersensitivity response within the nasal mucosa triggered by allergen exposure.^2^ Recent guidelines from the International Consensus statement on Allergy and Rhinology: Allergic Rhinitis 2023 (ICAR-Allergic Rhinitis 2023)^3^ and the British Society of Allergy and Clinical Immunology (BSACI)^1^ draw attention to contemporary strategies for diagnosing and managing AR. In this article, we review and generate evidence-informed recommendations for the management of AR in children and adults in family medicine.

## 2 Making A Diagnosis

A comprehensive diagnostic approach begins with a thorough history, enquiring about nasal symptoms of congestion and clear rhinorrhea, including onset, duration, triggers, and seasonal variation. Age of onset occurs predominantly in childhood and adolescence, and has been reported in children as young as 12 months.^3^ The onset of symptoms is often within minutes following exposure to allergens. Common triggers include aeroallergens such as tree pollen, grass pollen, house dust mites, molds, and animal dander.^3^ Other signs of AR include sneezing and an itchy nose, and if accompanied by allergic conjunctivitis: epiphora and ocular pruritus.^1,2,4^ Past medical history may include atopic conditions such as eczema and asthma. A family history of atopy is common. Evaluating the impact on the individual’s daily life, including sleep quality and school performance is particularly pertinent in the pediatric population.^3^

A general examination should inspect for stigmata of allergy, such as transverse dorsal nasal crease, open-mouth breathing, and allergic shiners (dark circles under the eyes). Anterior rhinoscopy can be performed by gently lifting the nasal tip upward while inspecting with an otoscope. We recommend a three-point inspection, looking at the anterior septum, inferior turbinate, and nasal floor. This examination aims to identify signs of inflammation: erythema, edema, septal deviation, polyps, hypertrophy, and pale blue discoloration of the inferior turbinate.^5^ The inferior turbinate (normal anatomical structure on the lateral nasal wall) can be mistaken for a polyp (benign growths extending from inflamed nasal mucosa). However, it is necessary to be able to clinically distinguish between the two to avoid unnecessary concern. A systematic approach is outlined in Table 1.

## 3 What Are The Differential Diagnoses?

The symptoms of AR are often nonspecific, making the differential diagnosis broad. These include viral upper respiratory tract infection; rhinitis medicamentosa (drug-induced rhinitis caused by prolonged use of over-the-counter nasal decongestants); occupational rhinitis; smoke-induced rhinitis; and vasculitis.^1–4^ Red flag symptoms that deviate from the typical AR presentation may raise suspicion of an alternative diagnosis: they are listed in Table 2.^1,3,4^

## 4 When Is Allergen Testing Indicated?

Practitioners should organize skin prick testing or serum allergen-specific IgE investigations when suspecting a diagnosis of AR. AR can be secondary to outdoor allergens usually linked to seasonal AR (few months a year during pollen season) and indoor allergens commonly linked with perennial AR (all year). Allergens responsible for seasonal AR include pollens from tree, grass, and weed; whereas, perennial AR is commonly triggered by dust mite, animal dander, and molds. Allergen testing plays a role in confirming diagnosis and provides important advice about allergen avoidance.^3^ Radiological imaging has no role in AR.^3^

## 5 Personalizing Treatment

Developing a treatment plan that is personalized first involves counseling patients that AR is a chronic condition, and that the treatment is focused on symptom control. Second is the implementation of allergen avoidance strategies. Patients can be directed to online information about allergen avoidance, for example, at Allergy UK. Third is to ensure the correct technique is followed when administering intranasal therapy. Intranasal saline is recommended as a component of the overall treatment strategy.^3^ Pharmacological treatment options include oral nonsedating antihistamines (e.g., cetirizine, loratadine, and fexofenadine), intranasal antihistamines (e.g., azelastine hydrochloride), and intranasal corticosteroids. Contrary to misconceptions, second-generation intranasal corticosteroids sprays (INCS) (e.g., Avamys, Nasonex, and Flixonase) have a bioavailability of <1% making them safe for long-term use.^6^

Routine use of oral decongestants (e.g., Pseudoephedrine hydro-chloride) is not recommended for AR.^3^ However, for short-term relief, a nasal decongestant (e.g., xylometazoline hydrochloride) can be used.^1,3,4^ Similarly, oral corticosteroids can be used for a brief duration,^1–4^ but clinical prescribers should weigh the potential systemic risks associated with their use. An increased risk of adverse effects (e.g., upper gastro-intestinal hemorrhage, sepsis, and heart failure) is observed even when oral steroids are used as infrequently as twice in a year.^7^

**Figure 1:**
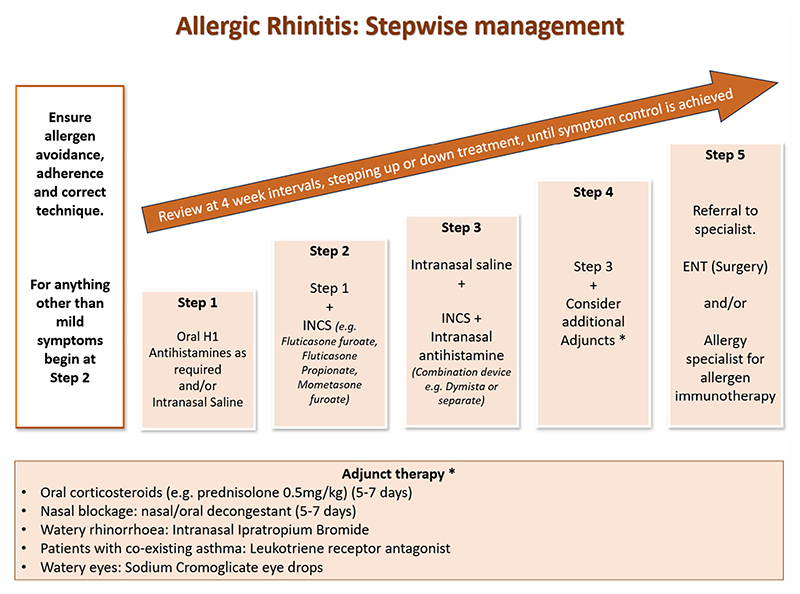
A stepwise approach to the management of AR in family medicine.

In adopting a systematic step-up and step-down approach, akin to the British Thoracic Society/Scottish Intercollegiate Guidelines Network asthma guidelines, we have produced a flowchart (Figure 1) summarizing the management of AR. Treatment responses should be evaluated within 2−4 weeks after commencement.^4^ Well-controlled AR can also help improve asthma control in those with coexistent disease.^8^

## 6 When To Refer For Specialist Care?

Consider urgent referral to an ear, nose, and throat (ENT) specialist if there are red flag features (Table 2). ENT surgical teams should be consulted in the presence of structural abnormalities that impair delivery of topical therapy, such as septal deviation or refractory hypertrophy of the inferior turbinates. In cases where symptoms persist despite optimal medical treatment, consider a referral to an allergy specialist for immunotherapy.

## 7 Summary

This article summarizes evidence-informed guidance for the diagnosis and management of AR for family medicine services. Our stepwise approach enables family medicine clinicians to provide patients with a personalized management plan and optimize treatment for AR.

## Figures and Tables

**Table 1:** Key characteristic differences between enlarged turbinates and nasal polyps.

	Examination	Enlarged turbinate	Nasal polyp
Inspection	Color/appearance	Pink	Pale/clear
Palpation with cotton-tipped	Consistency	Hard/fleshy	Soft/
swab			gelatinous
	Sensitivity to probing	Sensitive	Insensitive
	Mobility	Fixed	Mobile

**Table 2:** Red flags in patients presenting with nasal symptoms and associated differential diagnosis.

	Red flags	Differential diagnoses to consider
History	Unilateral nasal discharge/clear watery rhinorrhea	Foreign body nose; fungal ball; odontogenic sinusitis; tumor; CSF leak
	Facial pain	Tumor; chronic rhinosinusitis
	Altered smell	Tumor; chronic rhinosinusitis with polyposis
Examination signs	Unilateral polyp/nasal mass	Tumor including benign lesions (antro-choanal polyp, inverted papilloma, and extra nasopharyngeal angiofibroma) or malignancy
	Nasal crusting, contact bleeding, septal perforation, and oronasal fistula (perforation of hard palate)	Tumor, vasculitis, and granulomatous conditions
	Cheek swelling and infraorbital numbness	Tumor
	Polyps in children	Cystic fibrosis, primary ciliary dyskinesia, and immunodeficiency
	Orbital signs (proptosis and diplopia)	Tumor, sphenoiditis, and mucocele
	Cervical lymphadenopathy	Sinonasal malignancy

## References

[1] Scadding GK, Kariyawasam HH, Scadding G, Mirakian R, Buckley RJ, Dixon T (2017). BSACI guideline for the diagnosis and management of allergic and non-allergic rhinitis (Revised Edition 2017; First edition 2007). Clin Exp Allergy.

[2] BMJ Best Practice (2023). Allergic rhinitis.

[3] Wise SK, Damask C, Roland LT, Ebert C, Levy JM, Lin S (2023). International consensus statement on allergy and rhinology: allergic rhinitis – 2023. Int Forum Allergy Rhinol.

[4] National Institute for Health and Care Excellence (2024). Clinical knowledge summaries (CSK) − allergic rhinitis: management.

[5] Ziade GK, Karami RA, Fakhri GB, Alam ES, Hamdan AL, Mourad MM (2016). Reliability assessment of the endoscopic examination in patients with allergic rhinitis. Allergy Rhinol (Providence).

[6] Daley-Yates PT, Larenas-Linnemann D, Bhargave C, Verma M (2021). Corticosteroids: topical potency, systemic activity and therapeutic index. J Asthma Allergy.

[7] Yao TC, Huang YW, Chang SM, Tsai SY, Wu AC, Tsai HJ (2020). Association between oral corticosteroid bursts and severe adverse events: a nationwide population-based cohort study. Ann Intern Med.

[8] Brożek JL, Bousquet J, Agache I, Agarwal A, Bachert C, Bosnic-Anticevich S (2017). Allergic rhinitis and its impact on asthma (ARIA) guidelines-2016 revision. J Allergy Clin Immunol.

